# Robustness of the *Cupriavidus necator*-Catalyzed Production of α-Humulene

**DOI:** 10.3390/bioengineering12030323

**Published:** 2025-03-20

**Authors:** Lucas Becker, Emely Dietz, Dirk Holtmann

**Affiliations:** 1 Bioprocess Intensification, Institute of Bioprocess Engineering and Pharmaceutical Technology, Technische Hochschule Mittelhessen, 35390 Giessen, Germany; lucas.becker@lse.thm.de (L.B.); emely.dietz@lse.thm.de (E.D.); 2 Institute of Process Engineering in Life Sciences, Karlsruhe Institute of Technology, 76131 Karlsruhe, Germany

**Keywords:** process robustness, *Cupriavidus necator*, terpenes, α-humulene, preculture variation

## Abstract

The increasing global demand for natural substances such as the sesquiterpene α-humulene makes optimizing microbial production essential. A production process using the versatile host *Cupriavidus necator* has been recently improved by adjusting the minimal media and process parameters. Understanding microbial and process robustness is key to ensuring consistent performance under different conditions. This study is the first to investigate and quantify the robustness of microbial α-humulene production and biomass formation using *C. necator* pKR-hum. Established process improvements and the impact of common or individual precultures were analyzed and quantified for their effect on the robustness of product and biomass formation. We report a robust α-humulene production process with even more consistent biomass formation using *C. necator* pKR-hum. Even with a simulated process disturbance, 79% of the maximum α-humulene level was still produced. Overall, our results show that the α-humulene production process using *C. necator* pKR-hum is highly robust, demonstrating its resilience to process disturbances and suitability for further industrial applications.

## 1. Introduction

The microbial production of complex, bio-based compounds has gained significant attention due to its efficiency and versatility, and it is being increasingly adopted in industrial manufacturing processes. One of these natural compounds is α-humulene, a sesquiterpene found in high concentrations in plants like hops (*Humulus lupulus*), where it serves as a key aromatic compound [1]. α-Humulene has a broad range of applications, with uses in the food, fragrance, and cosmetics industries, and exhibits potential pharmaceutical properties such as anti-microbial [1], anti-tumor [2], and anti-inflammatory effects [3].

The microbial production of α-humulene offers several advantages over conventional terpene plant extraction or chemical synthesis methods, such as a higher yield, better controllability, improved cost-effectiveness, and the potential for process intensification to meet the growing global demand. The use of the polyhydroxybutyrate (PHB)-deficient and versatile *C. necator* PHB-4 strain, known for its ability to utilize a wide range of substrates—from CO_2_ to fructose and waste sources—enables both heterotrophic and chemolithoautotrophic fermentation processes [4]. However, robustness plays a critical role in fermentation and process intensification. Process robustness is defined by Olsson et al. as the ‘ability of a system to maintain unchanged performance when one or more perturbations occur’ [5].

Additionally, microbial robustness must be considered in relation to the production host, which ideally maintains consistent biomass and product formation rates despite process disturbances or changes [6].

A production method robust at both the process and microbial cell levels is essential for the efficient bioproduction of valuable natural substances. Understanding this is key to subsequent bioprocess intensification. Therefore, the robustness of microbial α-humulene production can be enhanced by adjusting or optimizing individual process parameters. To better understand how these factors influence the process robustness of α-humulene production using *C. necator* pKR-hum, in this study, we investigated different process conditions that were shown to affect biomass formation and α-humulene production. These conditions include optimizations that increase α-humulene production and biomass formation, as reported by Becker et al. in a previous study [7]. Key parameters tested include the concentration of iron (II) sulfate heptahydrate in the minimal medium, the concentration of the inducer L-rhamnose, and the process cultivation temperature. A 5-fold increase in the established iron (II) sulfate heptahydrate concentration (to 3.75 mg/L) led to increased *C. necator* biomass growth [7]. Additionally, increasing the L-rhamnose inducer concentration from 0.2% to 2% (*v*/*v*), along with decreasing the cultivation temperature 24 h after the induction step, resulted in higher α-humulene levels [7]. In addition to examining the effects of implementing individual process conditions on robustness against standard parameters, we also tested the potential impact of inoculating the main cultures from either common or individual precultures.

The robustness of α-humulene production and biomass formation (n = 6) in *C. necator* pKR-hum across various conditions was calculated according to Formula (1), adopted from Trivellin et al. [8]:

(1) $$R=-\frac{\sigma^{2}}{\overset{-}{x}}$$

When calculating robustness, both positive and negative deviations from the mean value ($\overset{-}{x}$) affect the robustness value (R) due to the variance (σ^2^). An R value of 0 indicates ideal robustness. However, the robustness value alone does not directly reflect process performance, although value ranges can be compared with each other. For example, Torello Pianale et al. showed that the glycerol yield (g/g) of *S. cerevisiae* CEN.PK113-7D and PE2 can be described with robustness values of −0.8 and −0.5, respectively, while the cell mass yield (g/g) for these strains was calculated as −0.6 and −0.4 [9]. These results suggest that cell mass formation is a more robust sub-process with fewer fluctuations than glycerol production in both strains.

In this study, we quantitatively assess how key process parameters affect the robustness of α-humulene production in *C. necator* pKR-hum. By comparing their effects, along with preculture handling, on the robustness of α-humulene and biomass formation, we provide insights for optimizing microbial processes in industrial applications. Understanding these factors is crucial for developing robust, scalable production systems for α-humulene and other valuable bio-based compounds using the versatile *C. necator* strain.

## 2. Materials and Methods

The materials and methods used in this study were partially adopted from Becker et al. [7]. The following sections describe the standard methodology and parameters for the fermentation experiments, which were consistently applied unless otherwise stated. Individual process condition changes as deviations from the standard run are noted in the Figure legends. The 2-phase temperature run included two cultivation temperature stages: 30 °C until 24 h after induction, followed by a switch to 25 °C. The final combination of all individually modified conditions was tested with 8 g/L D-fructose in addition to the standard 4 g/L as the substrate source. Additional robustness tests, simulating process disturbances with a 12 h shaking pause after the induction step, were performed to assess the robustness of the bacterial production host, *C. necator* pKR-hum.

### 2.1. Heterotrophic Cultivation of C. necator pKR-Hum

To ensure consistent starting conditions, *C. necator* pKR-hum cryo cultures were prepared and stored at −80 °C. Cells were grown in a lysogeny broth (LB) medium supplemented with 15 μg/mL tetracycline (TC) at 30 °C with shaking at 180 rpm (Ecotron, Infors HT; Bottmingen, Switzerland). After growth, the cells were centrifuged at 5000× *g* for 5 min and mixed with 25% (*v*/*v*) sterile glycerol for cryostorage.

For the experiments, 3 mL of the LB medium with TC was inoculated from a cryo culture and cultivated in a 15 mL sterile tube for 24 h as a preculture under the same conditions. *C. necator* pKR-hum main cultures (20 mL of minimal medium in 250 mL Erlenmeyer flasks) were then inoculated from either a common preculture (100 mL Erlenmeyer flask containing 10 mL of LB medium + TC) or from individual precultures (15 mL tubes with 3 mL of LB medium + TC) to investigate potential influences of preculture handling. The amount of biomass equivalent to an OD_600_ of 0.10 (0.043 g/L) was taken from the precultures, centrifuged, resuspended in 20 mL of the main culture medium with TC, and cultivated at 30 °C with 180 rpm. To visualize the growth curve, biomass was monitored every 60 s using the Cell Growth Quantifier device (CGQ, Scientific Bioprocessing; Pittsburgh, PA, USA). The recorded backscatter values were calibrated with offline OD_600_ measurements taken before starting and after the end of the cultivation.

### 2.2. Preculture and Main Culture Media

The standard composition of the minimal medium (MMasy) used in all the cultivations, based on Sydow et al. [10], is listed in Supplementary Materials—Tables S1 and S2. Stock solutions of the components were individually prepared with deionized water. After dissolving the components, all stock solutions, except for the trace element stock, were autoclaved and stored at room temperature. This minimal medium composition was used for all experiments unless stated otherwise due to additional supplementation with FeSO_4_·7H_2_O. The LB preculture media were prepared following standard recipes (see Supplementary Materials—Table S3).

### 2.3. C. necator pKR-Hum Based α-Humulene Production

*Cupriavidus necator* H16 PHB-4, a PHB-deficient strain, was used for microbial α-humulene production in this study. This strain was transformed through conjugation with the plasmid pKR-hum, which contains an L-rhamnose-inducible promoter, MVA pathway genes, and necessary genes for α-humulene production (*zssI* and *erg20*), along with a tetracycline resistance marker. For the pKR-hum plasmid map, refer to Supplementary Materials—Figure S1. Main cultures were induced at an OD_600_ of 0.50–0.70 (biomass 0.2–0.3 g/L) with 0.2% (*v*/*v*) L-rhamnose (unless otherwise specified) as the inducer to initiate α-humulene production, obtained by diluting the 20% (*w*/*v*) L-rhamnose stock solution 1:100. The stock solution was prepared in advance, sterile filtered, and stored at −20 °C. To facilitate the in situ product removal of the extracellularly released α-humulene, 20% (*v*/*v*) pure n-dodecane was added to the shake flasks immediately after the induction step [11]. A second phase formed above the cultivation broth, from which a control sample was taken directly after the addition of n-dodecane, and then a further sample was taken after 48 h.

### 2.4. α-Humulene Quantification

Due to α-humulene’s low water solubility, it was extracted and analyzed in the in situ n-dodecane phase. After centrifugation (5 min, 1000× g), 100 µL of the upper n-dodecane phase was stored in GC glass vials at −20 °C. Immediately before measurement, samples were diluted with 900 µL of acetone at a ratio of 1:10. Calibration curve standards were prepared from an α-humulene stock solution (83351, PhytoLab; Vestenbergsgreuth, Germany), diluted with pure acetone using the same method. The top standard was prepared at a concentration of 100 mg/L α-humulene. Final process levels measured 48 h after induction were converted relative to the aqueous phase. The detection of samples and standards was performed via gas chromatography–mass spectrometry (GC-MS; 7890B GC-MS with 5977B GC/MSD, Agilent Technologies; Santa Clara, CA, USA) with the N_2_ flow set at 45 mL/min, air flow set at 450 mL/min, and an FID temperature of 250 °C. Samples and standards were separated using an HP-5ms GC column (Agilent 19091S-433, Agilent Technologies; Santa Clara, CA, USA) and analyzed via the single-ion monitoring (SIM) method at an *m*/*z* ratio of 204.2 for α-humulene detection.

## 3. Results and Discussion

Subsequently, the robustness of α-humulene production using *C. necator* pKR-hum was evaluated, focusing on the influence of changes in previously established individual process factors. Factors such as the composition of the minimal medium, L-rhamnose induction concentration, and cultivation temperature were adopted from [7], and the resulting robustness was assessed. Additionally, the effect of a simulated process disturbance on the robustness of the production host *C. necator* pKR-hum was investigated.

### 3.1. Effect of Preculture Handling on Robustness of α-Humulene Production

**Figure 1:**
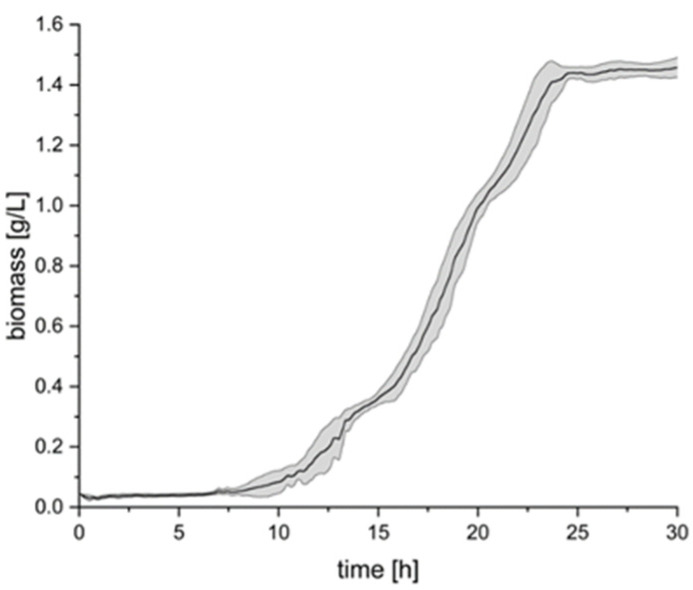
*C. necator* pKR-hum main culture growth in the minimal medium according to standard process conditions, with the standard media composition as described in Section 2.2, uninduced, at 30 °C with 180 rpm (n = 3).

A growth curve of the production strain *C. necator* pKR-hum was initially recorded in the minimal medium under standard process parameters, as seen in Figure 1.

Considering the growth curve in Figure 1, it can be deduced that the *C. necator* pKR-hum strain shows, as expected, rapid growth after the lag phase, reaching a stationary phase after 25 h at a biomass concentration of 1.45 g/L. This growth behavior was consistent across the tested flasks, with a small standard deviation (n = 3). In the following experiments, the effect of inoculating from a common LB medium preculture versus individual LB precultures (n = 6 each) on α-humulene production and robustness under varying process conditions using *C. necator* pKR-hum was investigated.

First, the influence of inoculating the main cultures from either a common LB preculture or individual LB precultures on final α-humulene levels and robustness was tested under standard conditions.

**Figure 2:**
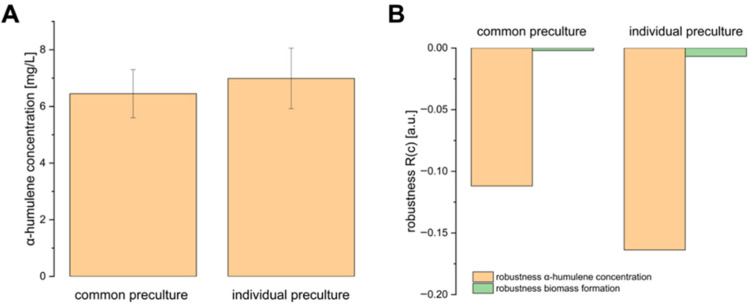
(**A**) α-Humulene level and (**B**) robustness of α-humulene production and biomass formation in *C. necator* pKR-hum, measured 48 h after induction, based on the standard process conditions: cultures were supplemented with 0.75 mg/L iron (II) sulfate heptahydrate and 4 g/L fructose, in accordance with the standard media composition in Section 2.2, incubated at 30 °C with 180 rpm, and induced with 0.2% (*v*/*v*) L-rhamnose at 0.2–0.3 g/L biomass. Comparison between main cultures inoculated either from a common LB preculture or from individual LB precultures (n = 6).

It is shown that, considering the standard deviations, there are no differences in the final α-humulene levels after 48 h of production between the cultures inoculated from a common LB preculture and individual LB precultures (Figure 2A).

The robustness of α-humulene production and biomass formation, whether inoculated from a common LB preculture or individual LB precultures, falls within a low robustness value range (Figure 2B), close to the ideal value of 0, with a maximum value of −0.16. This indicates a robust process under standard process conditions. However, the robustness values are lower and more robust when inoculating from a common LB preculture. Additionally, *C. necator* pKR-hum biomass formation is much more robust than α-humulene production, with robustness values of −0.002 and −0.007 compared to −0.11 and −0.16, respectively.

One possible explanation for this is the defined minimal medium used, which allows the biomass formation to adapt more consistently to the defined external conditions. In contrast, α-humulene production may fluctuate more between individual runs, potentially due to bacterial heterogeneities, such as varying gene expression cascades, metabolic burdens, or plasmid instability in the isogenic production cells [12,13].

### 3.2. Robustness of α-Humulene Production Under Varying Process Conditions

Next, the standard process parameters were extended with previously investigated, optimized, and established conditions, either individually or in combination. The *C. necator*-based α-humulene production was compared under these conditions, and their influence on the final α-humulene levels and robustness was tested.

**Figure 3:**
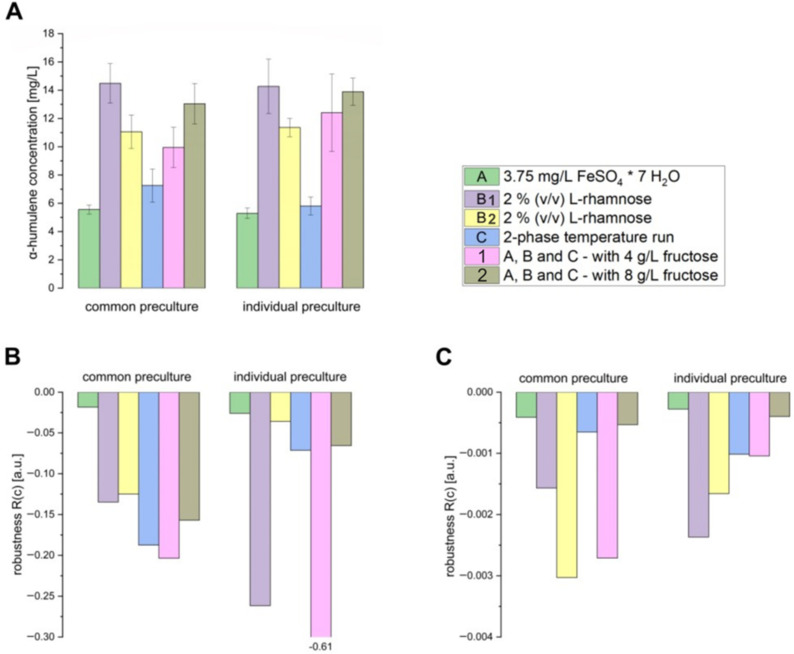
(**A**) α-Humulene level, (**B**) robustness of α-humulene production, and (**C**) robustness of biomass formation in *C. necator* pKR-hum 48 h after induction, based on the standard conditions listed below, each further extended with subsequent individual adjustments to process conditions: standard conditions with modification A—A 5-fold increase in the concentration of iron (II) sulfate heptahydrate (from 0.75 to 3.75 mg/L), standard conditions with modification B_1_—increasing the L-rhamnose inducer concentration from 0.2 to 2% (*v*/*v*), standard conditions with modification B_2_—repetition of B_1_, standard conditions with modification C—2-phase cultivation temperature, standard conditions with modifications 1—a combination of individual process conditions A, B, and C with 4 g/L fructose, standard conditions with modifications 2—a combination of individual process conditions A, B, and C with 8 g/L fructose. As standard conditions, the cultures were supplemented with 0.75 mg/L iron (II) sulfate heptahydrate and 4 g/L fructose, in accordance with the standard media composition in Section 2.2, incubated at 30 °C with 180 rpm, and induced with 0.2% (*v*/*v*) L-rhamnose at 0.2–0.3 g/L biomass. The 2-phase temperature run consisted of the following steps: 30 °C until 24 h after the induction step, followed by a decrease in the cultivation temperature to 25 °C. Comparison between main cultures inoculated either from a common LB preculture or from individual LB precultures (n = 6).

Figure 3A shows that the specific process conditions of 2% (*v*/*v*) L-rhamnose, a two-stage cultivation temperature, and the addition of 8 g/L fructose increased the final α-humulene levels after 48 h, despite the standard deviations. No differences in final α-humulene levels after 48 h of production were observed between cultures inoculated from either a common LB preculture or individual LB precultures. The robustness of both α-humulene production (Figure 3B) and biomass formation (Figure 3C) remained within a low robustness value range, regardless of whether inoculation was from a common LB preculture or individual LB precultures. This indicates robust implemented process conditions, regardless of preculture handling and inoculation. Moreover, this robustness towards both preculture handling variants also demonstrates the flexible and adaptable properties of the *C. necator* production strain, which are particularly in demand in industry and can reduce overall process complexity. However, the composition of the main medium can cause greater variation in α-humulene production by *C. necator*, with 2 mg/L α-humulene in grass juice medium and 10 mg/L α-humulene in LB medium being detected 42 h after induction [14]. Robustness towards variations in preculture handling is not always guaranteed and is often strain-specific. For example, in *E. coli*, population heterogeneities and fluctuating growth behavior as a result of variations in preculture conditions have been demonstrated by Hoang et al. [15]. Additionally, it can be seen that the robustness values of biomass formation lie in a much lower value range, suggesting more stable robustness than α-humulene production and indicating that essential and vital biological processes such as biomass formation in *C. necator* are more robust to external fluctuations and parameter variations than specific metabolic processes such as α-humulene production. Similar observations in *E. coli* were reported by Ishii et al., where enzyme levels were actively regulated to stabilize the metabolic status of the growth rate and, thus, the biomass formation for more robustness in the presence of fluctuations [16]. Overall, these results indicate highly robust product and biomass formation in *C. necator* pKR-hum, even under varying external process conditions such as the minimal medium composition or cultivation temperature.

### 3.3. Robustness of α-Humulene Production Following a Simulated Process Disturbance

Subsequently, the question arose as to how a simulated process disturbance would affect the robustness and final α-humulene level of the production process. To investigate this, a run combining conditions A, B, and C (3.75 mg/L FeSO_4_·7H_2_O, 2% (*v*/*v*) L-rhamnose, and a two-stage cultivation temperature with 8 g/L fructose) was simulated with a process disturbance. Therefore, the shaking was paused for 12 h following the induction step, with the culture held at 30 °C during this period.

**Figure 4:**
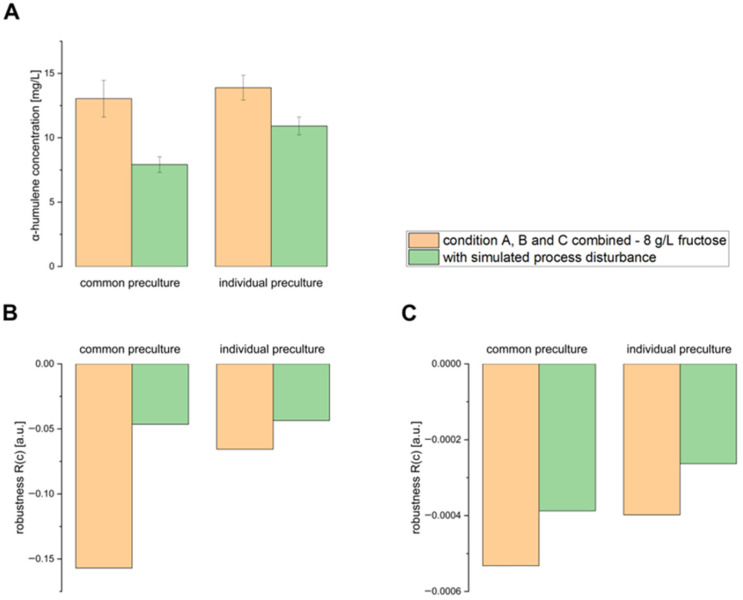
(**A**) α-Humulene level, (**B**) robustness of α-humulene production, and (**C**) robustness of biomass formation in *C. necator* pKR-hum 48 h after induction. The combined run included the following conditions: 3.75 mg/L FeSO_4_·7H_2_O, 2% (*v*/*v*) L-rhamnose, a 2-phase cultivation temperature, and 8 g/L fructose. The run was conducted both with and without a simulated process interruption, where shaking was paused for 12 h after the induction step while temperature control remained active. Cultures were induced at 0.2–0.3 g/L biomass. Comparison between main cultures inoculated either from a common LB preculture or from individual LB precultures (n = 6).

Figure 4A shows that there were no differences in the final α-humulene levels after 48 h of production when inoculations were performed from either a common LB preculture or individual LB precultures, taking standard deviations into account (with 8 g/L fructose in the combined run). A simulated process disturbance, created by interrupting shaking for 12 h after the induction step, reduced the final α-humulene levels after 48 h in both cases, regardless of how the precultures were handled. Still, the main cultures inoculated from individual LB precultures produced 79% of the maximum α-humulene level.

The robustness values for α-humulene production (Figure 4B) and biomass formation (Figure 4C) were low for both common and individual LB preculture inoculations, suggesting robust process conditions in the combined condition run with 8 g/L fructose. While both inoculation methods produced similar robustness values, inoculation from individual precultures resulted in slightly lower robustness values. Similarly to previous experiments, Figure 4B,C show that the biomass formation robustness values are lower than those for α-humulene production when testing the combined-condition run, indicating the highly robust biomass formation of *C. necator* pKR-hum.

The simulated process disturbance did not negatively impact the robustness of the production process, as the robustness values remained low, even with the process disturbance, indicating robust and continuous production by *C. necator* pKR-hum. Compared to the run without the simulated process disturbance, the final α-humulene levels reached 61% and 79% of the maximum after inoculation from common and individual precultures, respectively. One possible explanation for the high final α-humulene levels despite the process disturbances could be that *C. necator* is able to rapidly increase its flagella formation in response to nutrient and oxygen deficiencies caused by the lack of shaking. This enables even better motility and nutrient uptake in the unshaken culture, which, however, also consumes additional energy and could therefore have reduced α-humulene production. In the literature, this mechanism has been described by Parker et al. in poorly motile *E. coli* BW25113 cells, which became motile overnight after a shaking-free incubation in a liquid medium due to the upregulation of various motility genes [17].

## 4. Conclusions

Our study demonstrates that the α-humulene production process using *C. necator* pKR-hum with recently optimized parameters is highly robust. The robustness value for α-humulene levels under varying process conditions is −0.155 ± 0.143, while it is −0.002 ± 0.002 for biomass formation. Even with a simulated process disturbance, the process still produces 79% of the maximum α-humulene level compared to the undisturbed run, with a corresponding robustness value of −0.045 ± 0.001. These results indicate that the *C. necator*-based production process for α-humulene is robust to external fluctuations, which is crucial for large-scale industrial applications where such fluctuations may occur. Additionally, the lower robustness values for biomass formation as compared to α-humulene production also suggest that biomass formation is a more fundamental mechanism in *C. necator*. Contrary to this, the α-humulene biosynthesis route seems to be more sensitive to fluctuations, possibly due to the additional metabolic load involved in terpene production. Moreover, the α-humulene production process demonstrated robustness to inoculation from both common and individual precultures.

Overall, the quantification of robustness, along with these insights into both process and microbial robustness, contributes to a better understanding of the process for future industrial applications and supports the development of efficient terpene production processes. Future studies should concentrate on the optimization of existing metabolic pathways as well as the implementation of innovative genes for terpene production that also provide the maximum possible microbial and process robustness.

## Data Availability

The data presented in this study are available upon request from the corresponding author.

## Supplementary Materials

The following supporting information can be downloaded at https://www.mdpi.com/article/10.3390/bioengineering12030323/s1. Table S1: Standard composition of the minimal medium used (MMasy), with stock solutions corresponding to 10-fold, 100-fold, and 20,000-fold final medium concentrations; Table S2: Trace element composition of the minimal medium used (MMasy); Table S3: Composition of the lysogeny broth (LB) preculture medium; Figure S1: Plasmid map of the α-humulene production plasmid pKR-hum.
