# Supplementary material for: Robustness of the Cupriavidus necator-Catalyzed Production of α-Humulene
Source: Bioengineering (Basel). 2025 Mar 20;12(3):323. doi: 10.3390/bioengineering12030323 (PMC11939629; doi:10.3390/bioengineering12030323)
Supplement: Supplementary file 1 [file bioengineering-12-00323-s001.zip › bioengineering-3381627-supplementary.pdf]

Supplementary materials

Robustness of the *Cupriavidus necator*-Catalyzed Production of α-humulene

Lucas Becker [1], Emely Dietz [1] and Dirk Holtmann [2,*]

[1] Bioprocess Intensification, Institute of Bioprocess Engineering and Pharmaceutical Technology, Technische Hochschule Mittelhessen, 35390 Giessen, Germany; lucas.becker@lse.thm.de (L.B.); emely.dietz@lse.thm.de (E.D.)
[2] Institute of Process Engineering in Life Sciences, Karlsruhe Institute of Technology, 76131 Karlsruhe, Germany
* Correspondence: dirk.holtmann@kit.edu

The following two Tables S1 and S2 list the standard composition of the minimal medium (MMasy) and trace elements used in this study, based on Sydow et al. (2017). Tables S1 – S3 and Figure S1 adopted from: Becker, L.; Dietz, E.; Holtmann, D. Individual process steps optimization of *Cupriavidus necator*-catalyzed production of α-humulene. Biochemical Engineering Journal, 109617. 2024. https://doi.org/10.1016/j.bej.2024.109617.

Table S1. Standard composition of the minimal medium used (MMasy), with stock solutions corresponding to [1)] 10-fold, [2)] 100-fold, and [3)] 20,000-fold final medium concentrations.

**Media component**		**Medium concentration [g/L]**
Na_2HPO_4	1)	2.895
$NaH_2PO_4 * H_2O$	1)	2.707
K_2SO_4	1)	0.170
$CaSO_4 * 2\ H_2O$	1)	0.097
$MgSO_4 * 7\ H_2O$	2)	0.800
$(NH_4)_2SO_4$	2)	0.943
Trace elements	3)	1:20,000 from stock
D-fructose	2)	4.0

Trace elements were added to the medium at the concentrations listed in Table S2. To prepare the trace element stock solution, the components were dissolved in 0.1 M

hydrochloric acid at 20,000 times the final medium concentration, then sterile filtered. The stock solution was stored at 4 °C until use in the minimal medium.

Table S2. Trace element composition of the minimal medium used (MMasy).

Trace element component	Medium concentration [µg/L]
$FeSO_4 * 7 H_2O$	750
$MnSO_4 * H_2O$	120
$ZnSO_4 * 7 H_2O$	120
$CuSO_4 * 5 H_2O$	24
$Na_2MoO_4 * 6 H_2O$	90
$NiSO_4 * 6 H_2O$	75
$CoSO_4 * 7 H_2O$	2

The following Table S3 lists the standard composition of the lysogeny broth (LB) preculture medium used in this study.

Table S3. Composition of the lysogeny broth (LB) preculture medium.

Media component	Concentration [g/L]
Yeast extract	5
Tryptone	10
NaCl	5

The following plasmid map in Figure S1 shows the pKR-hum expression plasmid used, which was transformed into *C. necator* H16 PHB-4 for α-humulene production. This plasmid contains a tetracycline resistance gene, the α-humulene synthase gene (*zzsI*), the farnesyl pyrophosphate synthase gene (*erg20*), and the MVA pathway enzyme genes: *hmgs*, *fni*, *hmgr*, *mvaK*, *mvaD*, and *mvaK2*. In addition to *C. necator*-based α-humulene production, other potential production hosts exist for the α-humulene biosynthesis. Yeasts, for example, are a well-researched group. Compared to these, the *C. necator*-based batch production system using shake flasks operates at lower final α-humulene titers. For example, Guo et al. (2022) were able to produce up to 21.7 g/L of α-humulene using a fed-batch bioreactor system combined with an engineered *Y. lipolytica* yeast strain. This titer once again exceeds the highest α-humulene titer previously achieved with *C. necator*, which was 2 g/L, as reported by Milker et al. (2021).

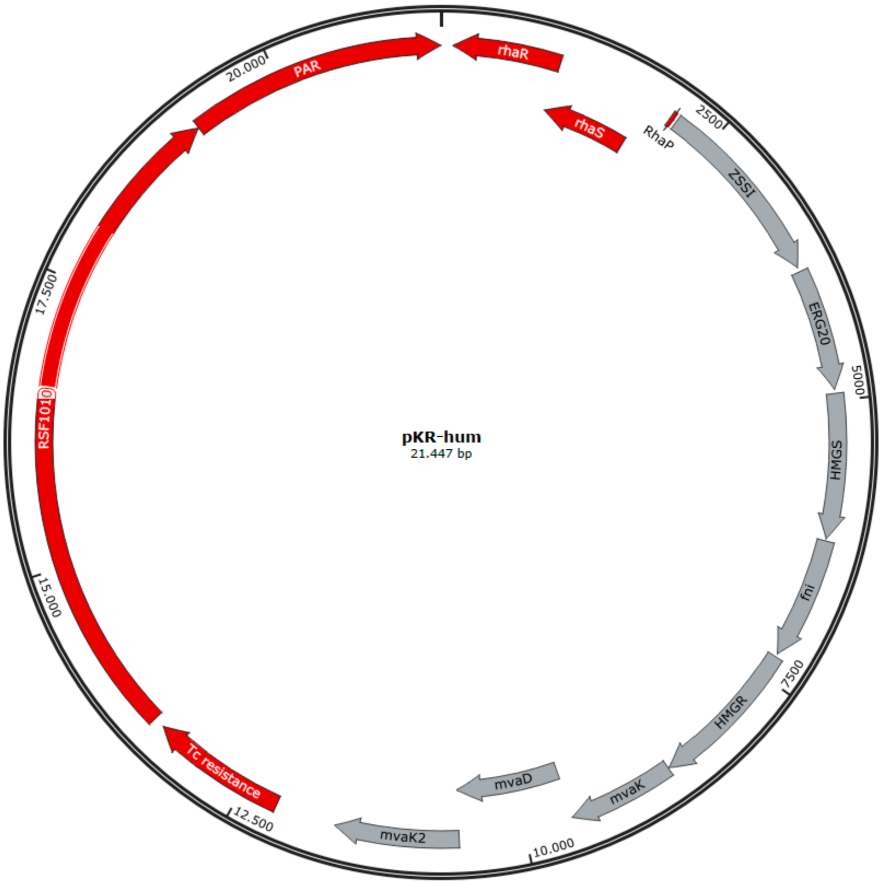

Figure S1. Plasmid map of the α-humulene production plasmid pKR-hum.
